# The cross-city mobility patterns and incubation period of the early-stage chikungunya fever outbreak in Guangdong Province, China, in 2025

**DOI:** 10.1371/journal.pntd.0014360

**Published:** 2026-05-22

**Authors:** Jianhua Huang, Jinyu Huang, Lihan Liang, Ting Hu, Dejian Zhao, Yingtao Zhang, Yue Zhao, Siyang Jiang, Guoren Wang, Jiahui Liu, Yali Zhuang, Meng Zhang, Jie Huang, Shuwen Dong, Xiqing Huang, Ming Kang, Wenjia Liang, Yan Li

**Affiliations:** 1 Guangdong Center for Disease Control and Prevention, Guangzhou, Guangdong, China; 2 Guangzhou Center for Disease Control and Prevention, Guangzhou, Guangdong, China; 3 Foshan Center for Disease Control and Prevention, Foshan, Guangdong, China; Faculty of Science, Ain Shams University (ASU), EGYPT

## Abstract

**Background:**

Chikungunya fever (CF), an arthropod-borne disease caused by Chikungunya virus (CHIKV), is a serious public health threat globally. In July 2025, an outbreak occurred in Foshan City, Guangdong Province, China. A large number of these cases involved cross-city movements, which complicated containment efforts and provided a unique opportunity to study transmission patterns and key epidemiological parameters.

**Methods:**

We obtained information on 400 confirmed cases of CF with cross-city exposure histories reported across Guangdong Province in southern China by 21 August 2025. Demographic, clinical, and mobility data were mainly obtained from the National Notifiable Infectious Disease Reporting System and supplemented by epidemiological investigations. The incubation period was estimated using a parametric accelerated failure time model, with log-normal, gamma, Weibull, and Erlang distributions used to fit the model. Subgroup analysis was performed based on mobility patterns and exposure windows.

**Principal findings:**

Significant demographic differences were observed compared with local Foshan cases: cross-city cases had higher proportions of males (58.3%), individuals within the age groups of 15–24, 25–34, and 35–44,and individuals with occupations of general staff/workers, business/service workers, and students. Fever (86.4%), arthralgia (79.3%), and rash (61.2%) were the most common symptoms. Mobility analysis revealed that Foshan and Guangzhou were the major sources of infection, with cases spreading mainly to cities within the Pearl River Delta and provinces such as Guangxi (43.3%) and Hunan (15.4%). The median incubation period was estimated to be 5.4 days (95% CI 5.0–5.7), with 2.5th and 97.5th percentiles of 2.5 days and 11.4 days, respectively.

**Conclusions:**

This study underscores the central role of population mobility in the spread of CHIKV and highlights distinct epidemiological characteristics of cross-city cases. The estimated median incubation period of 5.4 days provides important evidence for surveillance and response strategies during chikungunya outbreaks. Notably, students and migrant workers accounted for a higher proportion of cross-city cases, suggesting that highly mobile populations may contribute to inter-regional transmission. These findings highlight the importance of strengthened surveillance and coordination across regions for the prevention and control of future outbreaks in Guangdong and other high-risk areas in China.

## Author summary

In this study, we investigated the Chikungunya fever outbreak that occurred in 2025 in Guangdong Province, China, focusing on cases that spread across different cities. By analyzing 400 cross-city cases, we identified that the key demographic groups at higher risk of spreading the virus comprise young adults, students, and migrant workers. We traced how population mobility contributed to the outbreak beyond its origin. We also estimated the median incubation period to be 5.4 days, with 98.2% (95%CI 96.7%–99.3%) symptomatic cases developing illness within 12 days of exposure. These findings emphasize the importance of tailoring public health measures to mobile populations and support the current 12-day monitoring policy for exposed individuals. This study provides practical evidence to help prevent and control future outbreaks of mosquito-borne diseases in regions experiencing high population movement.

## Introduction

Chikungunya virus (CHIKV), first identified in Tanzania in 1952 [1], is an enveloped, positive-sense, single-stranded RNA virus belonging to the genus *Alphavirus* within the family Togaviridae. It causes Chikungunya fever (CF), an arthropod-borne disease transmitted primarily through the bites of infected *Aedes aegypti* and *Aedes albopictus* mosquitoes. Most CF patients develop acute symptoms, notable for the rapid onset of fever, incapacitating polyarthralgia, arthritis, rash, myalgia, and headache [2]. Arthralgia may persist for months or even longer in more than 30% of patients [3].

Climate change, globalization, and urbanization contribute to the risk associated with the global epidemic of CHIKV [4]. Key genetic changes, such as the E1-A226V and E2-L210Q mutations, have improved the adaptability of the virus to *A. albopictus* mosquitoes. A more suitable climate for mosquitoes can boost their reproduction and vectorial capacity, thereby expanding the potential range of CHIKV transmission [5]. To date, CHIKV has been documented in over 110 countries [6] and has caused two global pandemics: the Indian Ocean pandemic in 2004–2005, driven by the East-Central-South African (ECSA) genotype, and the American pandemic in 2013–2015, caused by the Asian genotype [7,8]. As CHIKV is a growing threat, it was added to the WHO Blueprint list of priority pathogens in July 2024 and was assessed as posing a high risk of triggering a Public Health Emergency of International Concern (PHEIC) [9].

In July 2025, an outbreak of CF occurred in Shunde District, Foshan City, Guangdong Province, China. The disease rapidly increased and spread beyond the district to other cities [10]. By September 1, over 10,000 confirmed cases were reported in Guangdong Province. A significant proportion of these cases involved cross-city migrants, whose constant movement across cities made it difficult to prevent and control the disease. Population mobility is highly responsible for spreading tropical epidemics and many existing empirical studies have confirmed its role in the prevalence and spread of epidemics [11]. Therefore, in this study, we performed epidemiological analysis of cross-city cases to derive evidence-based recommendations for preventing and controlling CF more effectively. The mobility of these individuals provides a unique opportunity to more accurately estimate the incubation period. As individuals were probably infected only during travel to affected areas, their well-documented movement timelines allow us to calculate the exposure windows more precisely [12]. Genomic sequencing confirmed that the outbreak in Foshan was caused by a viral strain belonging to the Central African Clade of ECSA genotype [13]. However, our understanding of the incubation period for this specific clade is limited, and whether new mutations have altered its infectiousness or natural history remains unclear. Previous studies on incubation period of CHIKV have been constrained by insufficient sample sizes and rough estimated time intervals, and have yielded inconsistent estimates [14–16]. To resolve these issues, we leverage comprehensive data from cross-city cases to characterize their epidemiological characteristics and model the incubation period using a parametric accelerated failure time approach. We provided valuable information on CHIKV transmission dynamics that can help in developing effective prevention strategies in mobile populations.

## Methods

### Ethics statement

This study was conducted in accordance with the Declaration of Helsinki and received ethical approval from the Medical Research Ethics Review Committee of Guangdong Provincial Center for Disease Control and Prevention (Ethics Review Number: W96-027E-202517). Due to the retrospective nature of the study and the use of de-identified data from notifiable infectious disease surveillance systems and local CDC reports, the requirement for informed consent was waived by the ethics committee.

### Data source

Data on CF cases in Guangdong Province were collected from July 8, 2025 (the date of the first reported case) to August 21, 2025. The most probable city of infection was determined based on detailed epidemiological investigation reports. Two trained field epidemiologists independently reviewed the residence, travel history, and exposure timeline of each case, and discrepancies were resolved by consensus. The likely place of infection was inferred according to the local transmission context and travel history. In general, infection was attributed to locations with documented ongoing transmission during the exposure period. If a case had stayed in multiple locations, the most probable location of exposure was determined based on the timing of travel relative to the epidemic activity. For cases with repeated travel between areas with and without reported transmission, the exposure window was defined as the period from the first arrival in a high-transmission area to the last departure from that area. Cases for which the infection location could not be reasonably inferred due to multiple concurrent exposure locations were excluded from analyses requiring identification of the infection site.

Cases were classified as cross-city infections when the inferred city of infection differed from the reporting location. Cross-city cases were further categorized as intra-provincial when they were reported within Guangdong Province and inter-provincial when they were reported outside the province.

For intra-provincial cases of CF, demographic characteristics (gender, age, and occupation) and the time of diagnosis were extracted from the National Notifiable Infectious Disease Reporting System (NNDRS), which is a real-time, internet-based surveillance system covering mainland China [17,18]. Additional details, including source area of infection, affected area, reason of exposure, exposure timelines (first and last), symptom onset time, and clinical manifestations, were obtained from epidemiological investigation reports issued by the Centers for Disease Control and Prevention (CDCs) of local cities. All data on inter-provincial cases of CF were extracted from the epidemiological investigation reports. All diagnosed cases of CF included in this study were confirmed by reverse transcription polymerase chain reaction (RT-PCR) [19]. Each case was independently double-entered by two investigators, with discrepancies resolved through discussion and consensus.

### Epidemiological and mobility patterns of the cross-city cases in this CF outbreak

We analyzed the epidemiological characteristics of the cross-city cases by delineating the demographic profiles and spatial mobility patterns. Key epidemiological features of cross-city cases were summarized by gender, age and occupation. Corresponding incidences of symptom, including rash, arthralgia, fever, diarrhea, vomiting, headache or dizziness, and ocular symptoms, were calculated. Comparisons with early local cases reported in Foshan highlighted distinct characteristics of cross-city cases [20]. To conduct a temporal analysis, we constructed an epidemic curve based on the dates of diagnosis to visualize the progression of cross-city spread. The mobility patterns of intra-provincial or inter-provincial mobile cases were further investigated using a geospatial sankey diagram to illustrate the geographic transmission of mobile cases across cities.

### Incubation period estimation

We applied a previously described parametric accelerated failure time model to estimate the incubation period [21]. Based on existing studies of CF and other mosquito-borne diseases such as dengue, we assumed that the incubation time of CF follows a log-normal distribution [16,22–25]. This primary model was used to fit all cross-city cases, and cases with exposure times under 24 h were extracted for subset analysis. The inter-provincial and intra-provincial cases were fit to the primary model individually. In a secondary analysis, three other common incubation time distributions (gamma, Weibull, and Erlang) were similarly fitted to the model. For each distribution, the median incubation period and significant quantiles (2.5th, 25th, 75th, and 97.5th percentiles) along with their bootstrapped confidence intervals (CIs) were calculated.

### Statistics and other analyses

Continuous variables were presented as the mean ± standard deviation (mean ± SD), median, and interquartile range (IQR), whereas categorical variables were presented as percentages. The analysis of variance (ANOVA) was used for age as a continuous variable, and the Kruskal-Wallis test was used for age as an ordinal categorical variable. Pearson’s Chi-square test was conducted to compare demographic characteristics, including sex, age group, occupations, reason of exposure and symptom incidences, and for the few categories, Fisher’s exact test was used instead. Multiple comparisons were corrected using the Bonferroni method. All results were considered to be statistically significant at *P <* 0.05 (two-sided). All statistical analyses were performed in the R software (Version 4.2.0; R Foundation for Statistical Computing, Vienna, Austria). The analyses involving the incubation period were conducted using the “coarseDataTools” and “activemonitr” packages in the R software. All the plots were generated using the R package “ggplot2”.

## Results

### Demographic distribution

Based on a comprehensive review of all documented cases in Guangdong Province by 21 August 2025, we identified a final cohort of 400 confirmed cases of CF with cross-city transmission links to Guangdong (Table 1). Among them, 296 cases moved within Guangdong (intra-provincial), whereas 104 cases showed movement between (inter-provincial) (Table 2). Among all cases, 167 were female (41.8%) and 233 were male (58.3%). Sex distribution differed slightly between groups: males constituted 60.1% of intra-provincial cases and 52.9% of inter-provincial cases. The mean age of all cases was 35.7 years (SD = 17.2), with intra-provincial cases averaging 35.1 years (SD = 17.0) and inter-provincial cases averaging 37.3 years (SD = 17.8). Cases aged 25–44 years accounted for more than 40% of the total cases. Occupational profiles were considerably different, with the most common occupations being general staff/workers, business/Service workers, and students; and students were most abundant among inter-provincial cases. Intra-provincial exposure was mainly attributed to local residence or work, while inter-provincial exposure was mainly attributed work or visiting relatives. Most cases presented with fever (86.4%), arthralgia (79.3%), and rash (61.2%), while fewer cases reported headache/dizziness (14.4%), ocular symptoms (1.5%), diarrhea (1.0%), and vomiting (0.5%).

**Table 1:** Characteristics of cross-city cases with CF^a^.

Characteristic	Cross-city cases, N = 400 (100%)
Gender, n(%)	
Female	167 (41.8%)
Male	233 (58.3%)
Age(years)	35.7 ± 17.2
Age group	
< 5	9 (2.3%)
5–14	48 (12.0%)
15–24	51 (12.8%)
25–34	83 (20.8%)
35–44	84 (21.0%)
45–54	63 (15.8%)
55–64	43 (10.8%)
≥ 65	19 (4.8%)
Occupation, n(%)	
Business/Service workers	71 (18.5%)
Homemakers/Unemployed	62 (16.2%)
Retirees	17 (4.4%)
Students	67 (17.5%)
General staff/workers	96 (25.1%)
Children	12 (3.1%)
Farmers	23 (6.0%)
Others	35 (9.1%)
Exposure reason, n(%)	
Local resident	108 (27.0%)
Work	128 (32.0%)
Visiting relatives	81 (20.3%)
Travel	41 (10.3%)
Other/Unknown	42 (10.5%)
Rash, n(%)	243 (61.2%)
Arthralgia, n(%)	315 (79.3%)
Fever, n(%)	343 (86.4%)
Diarrhea, n(%)	4 (1.0%)
Vomiting, n(%)	2 (0.5%)
Headache/Dizziness, n(%)	57 (14.4%)
Ocular symptoms, n(%)	6 (1.5%)

^a^CF = Chikungunya fever. Mean ± standard deviation for continuous variables; n (%) for categorical variables. Occupation were calculated based on cases with available occupation data (N = 383).

**Table 2:** Comparison of characteristics between intra-provincial and inter-provincial cases with CF^a^.

	Case classification		OR (95% CI)	*P* value
Characteristic	Intra-provincial, N = 296 (74%)	Inter-provincial, N = 104 (26%)		
Gender, n(%)			0.74 (0.48,1.17)	0.197
Female	118 (39.9%)	49 (47.1%)		
Male	178 (60.1%)	55 (52.9%)		
Age(years)	35.1 ± 17.0	37.3 ± 17.8	/	0.253
Age group			/	0.111
< 5	6 (2.0%)	3 (2.9%)		
5–14	36 (12.2%)	12 (11.5%)		
15–24	39 (13.2%)	12 (11.5%)		
25–34	68 (23.0%)	15 (14.4%)		
35–44	61 (20.6%)	23 (22.1%)		
45–54	46 (15.5%)	17 (16.3%)		
55–64	26 (8.8%)	17 (16.3%)		
≥ 65	14 (4.7%)	5 (4.8%)		
Occupation, n(%)			/	0.002
Business/Service workers	58 (20.1%)	13 (13.8%)		
Homemakers/Unemployed	51 (17.6%)	11 (11.7%)		
Retirees	11 (3.8%)	6 (6.4%)		
Students	47 (16.3%)	20 (21.3%)		
General staff/workers	80 (27.7%)	16 (17.0%)		
Children	8 (2.8%)	4 (4.3%)		
Farmers	17 (5.9%)	6 (6.4%)		
Others	17 (5.9%)	18 (19.1%)		
Exposure reason, n(%)			/	<0.001
Local resident	89 (30.1%)	19 (18.3%)		
Work	90 (30.4%)	38 (36.5%)		
Visiting relatives	46 (15.5%)	35 (33.7%)		
Travel	35 (11.8%)	6 (5.8%)		
Other/Unknown	36 (12.2%)	6 (5.8%)		
Rash, n(%)	167 (57.0%)	76 (73.1%)	0.48 (0.29,0.78)	0.004
Arthralgia, n(%)	223 (76.1%)	92 (88.5%)	0.40 (0.21,0.77)	0.008
Fever, n(%)	254 (86.7%)	89 (85.6%)	1.02 (0.54,1.93)	0.776
Diarrhea, n(%)	2 (0.7%)	2 (1.9%)	0.35 (0.05,2.50)	0.282
Vomiting, n(%)	1 (0.3%)	1 (1.0%)	0.35 (0.02,5.63)	0.456
Headache/Dizziness, n(%)	39 (13.3%)	18 (17.3%)	0.73 (0.39,1.33)	0.318
Ocular symptoms, n(%)	5 (1.7%)	1 (1.0%)	1.77 (0.20,15.33)	>0.999

^a^CF = Chikungunya fever. Mean ± standard deviation for continuous variables; n (%) for categorical variables. Occupation were calculated based on cases with available.

To further evaluate the demographic characteristics of cross-city cases in this epidemic, we compared the cross-city cases with early local cases in Foshan. Among cross-city cases, we observed a higher proportion of individuals aged within the age group of 15–24, 25–34 and 35–44 (all Chi-square test; *P <* 0.05; S1 Table), and a lower proportion of those who were 65 years old or older (Chi-square test; *P <* 0.001; S1 Table). Males were significantly over-represented (Chi-square test; *P =* 0.009), especially among intra-provincial cases. Moreover, students and general staff/workers accounted for a higher proportion of cases (Chi-square test; *P <* 0.001; S1 Table), whereas business/service workers, housekeepers/unemployed individuals and retirees accounted higher in the early cases in Foshan (Chi-square test; *P <* 0.001; S1 Table).

### Mobility patterns of the cross-city CF cases

**Fig 1:**
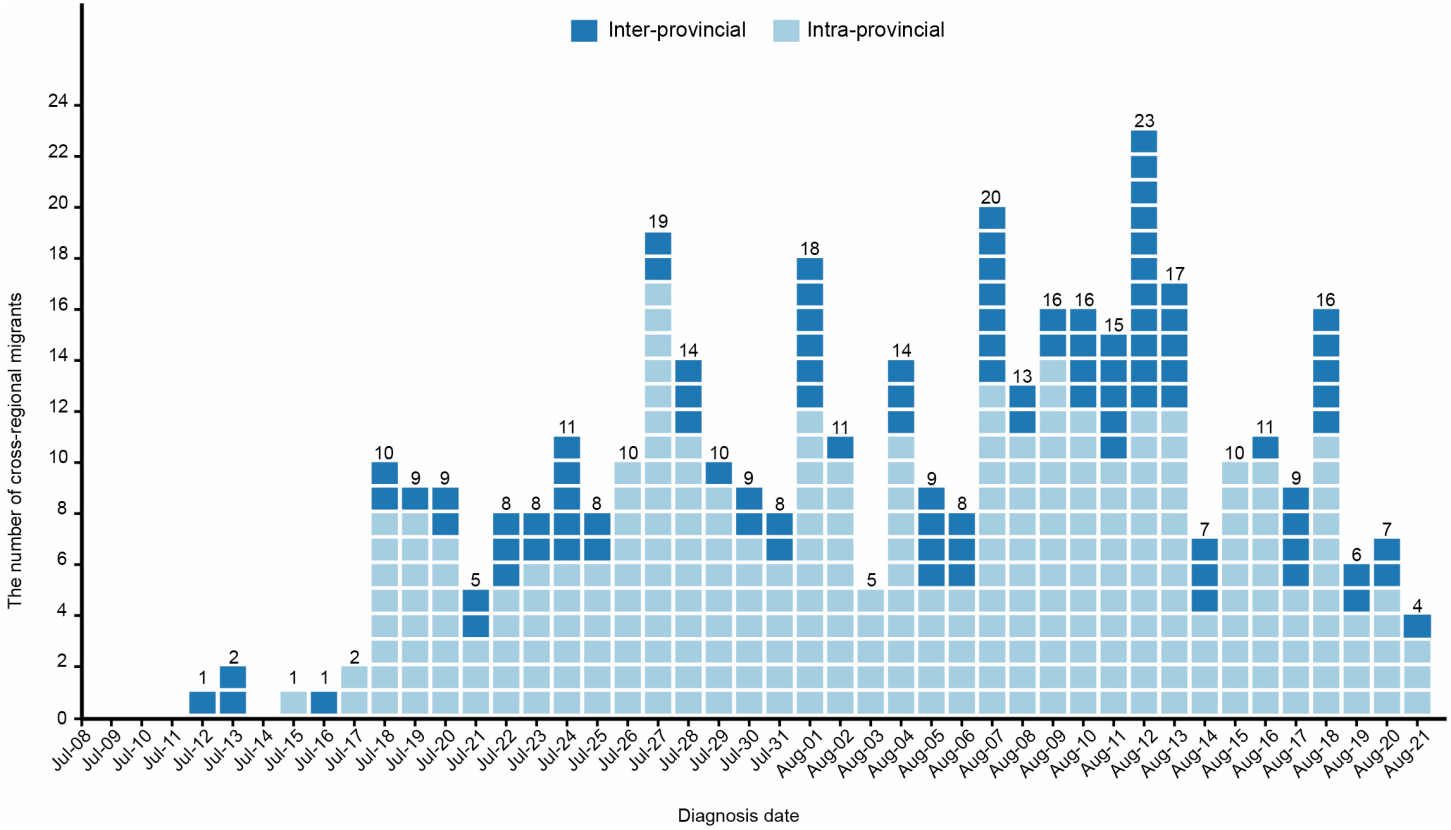
Time distribution of the cross-city cases of CF in Guangdong Province until August 21, 2025. The figure shows the daily reported cross-city cases from the beginning of the CF outbreak to August 21. The squares represent the number of cases and different colors are used to distinguish intra-provincial cases and inter-provincial cases. Data were collected up to August 21, 2025.

Since the onset of the outbreak of CF in Foshan, the daily increase in cross-city cases showed a fluctuating upward trend, reaching their peak on 12 August with a maximum of 23 cases before gradually declining thereafter (Fig 1).

**Fig 2:**
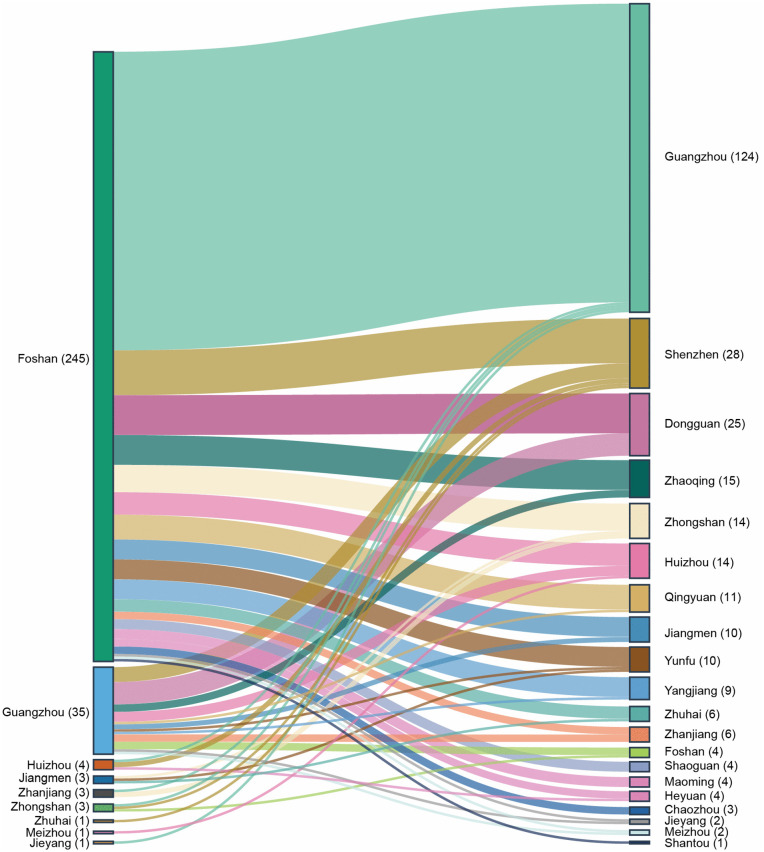
Migration patterns of intra-provincial cases of CF within Guangdong Province. The length of the colored blocks is proportional to the number of intra-provincial cases, and the lines indicate the direction of migration. The text in the figure refers to the names of municipal administrative regions.

For the intra-provincial cases, nine cities served as the source of infection and Foshan was the most frequent source, comprising 245 cases (82.8%), followed by Guangzhou with 35 cases (11.8%). A total of 20 cities were affected, primarily those surrounding Foshan, including Guangzhou (41.9%), Shenzhen (9.5%), Dongguan (8.4%), Zhaoqing (5.1%) and Zhongshan (4.7%) (Fig 2).

**Fig 3:**
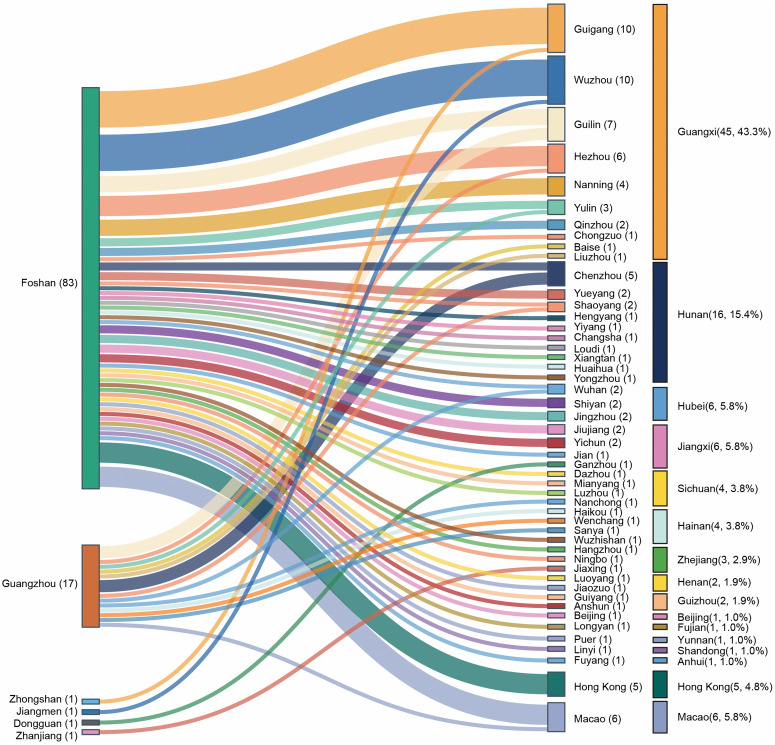
Migration patterns of CF inter-provincial cases from Guangdong Province to other provinces. The length of the colored blocks is proportional to the number of inter-provincial cases, and the lines indicate the direction of migration. The text in the figure refers to the names of municipal administrative regions.

For the inter-provincial cases, seven cities served as the source of infection, with most cases originating from Foshan (83 cases) and Guangzhou (17 cases). These cases spread across 49 destination cities, involving 14 provincial-level regions by 21 August, largely within Guangxi (45 cases; 43.3%) and Hunan Province (16 cases; 15.4%), which are important sources of labor for Guangdong Province. Additionally, five cases (4.8%) spread to Hong Kong and six cases (5.8%) to Macao (Fig 3).

### Estimation of the incubation period

**Fig 4:**
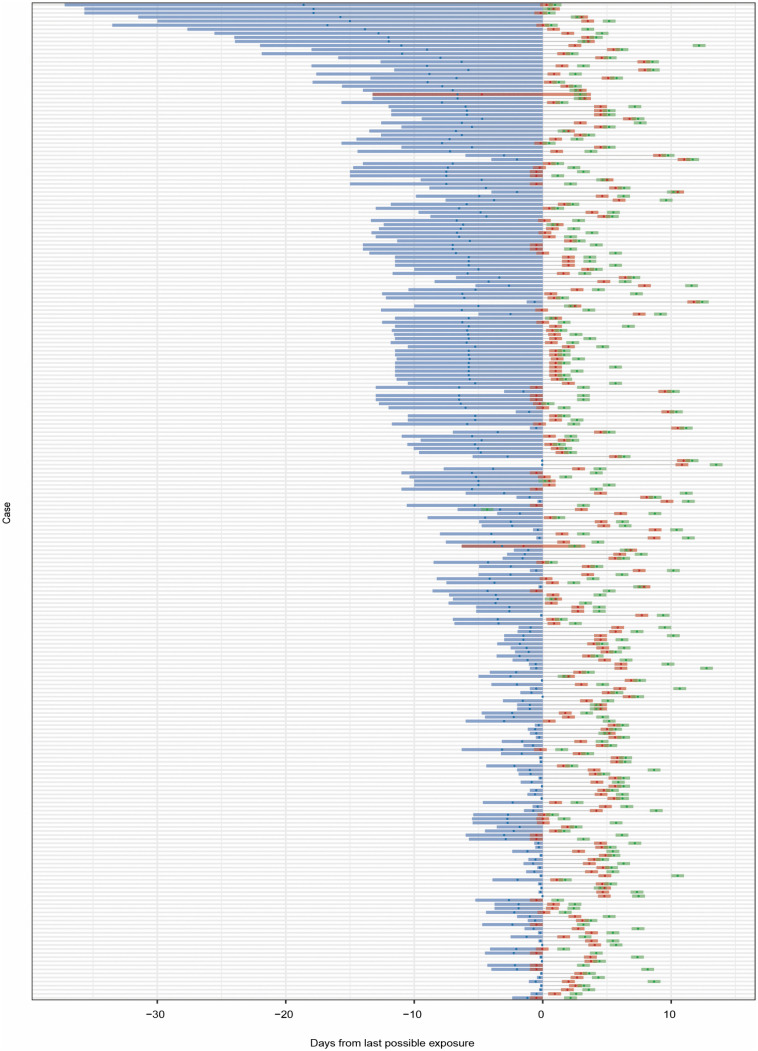
Chikungunya fever exposure (blue), symptom onset (red), and case diagnosis (green) times for 400 confirmed cases. Colored regions represent the full possible time intervals for exposure, symptom onset, and case diagnosis; points represent the midpoints of these intervals.

**Fig 5:**
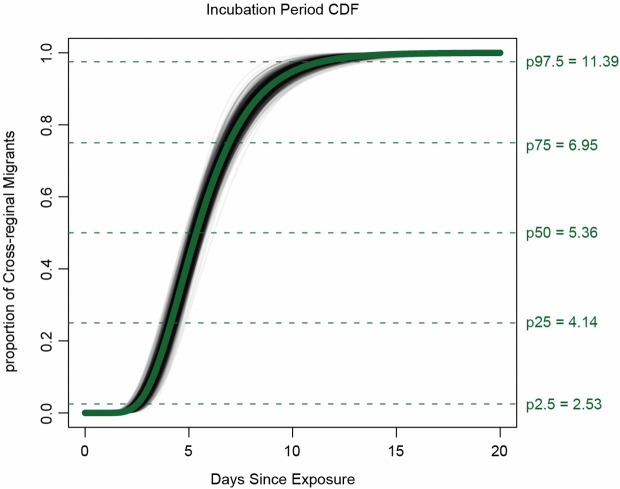
Cumulative distribution function of the CF incubation period estimate from the log-normal model. The estimated median incubation period of CF was 5.4 days (95% CI 5.0–5.7). We estimated that fewer than 2.5% of infected persons will display symptoms within 2.5 days (95% CI 2.2–2.9) of exposure, whereas symptom onset will occur within 11.4 days (95% CI 10.1–12.7) for 97.5% of infected persons. Horizontal bars represent the 95% CIs of the 2.5th, 50th, and 97.5th percentiles of the incubation period distribution. The estimate of the dispersion parameter is 1.47 (95% CI 1.39–1.54).

Through a comprehensive review of epidemiological investigations and the NNDRS, we constructed a timeline of exposure, symptom onset, and diagnosis for all 400 cross-city cases (Fig 4). Fitting the log-normal model to all cross-city cases, we estimated the median incubation period of CF to be 5.4 days (95% CI 5.0–5.7) (Fig 5). Fewer than 2.5% of cases were estimated to show symptoms within 2.5 days (95% CI 2.2–2.9) of exposure, and symptom onset will occur within 11.4 days (95% CI 10.1–12.7) for 97.5% of cases. The estimate of the dispersion parameter was 1.47 (95% CI 1.39–1.54), with a mean incubation period of 5.4 days. To control for potential bias from long-time exposure windows, we performed the same analysis on a subset of cases with known exposure time under 24 hours (n = 42). This yielded a similar median incubation period of 5.4 days (95% CI 4.8–6.1), with 2.5% of cases developing symptoms within 2.4 days (95% CI 1.9–3.2) and 97.5% within 11.9 days (95% CI 9.5–14.2) of exposure.

Because there are multiple potential transmission sources within Guangdong Province, the exposure histories of part intra-provincial cases were difficult to define precisely, leading to greater uncertainty in their exposure windows. In contrast, for inter-provincial cases (n = 104), exposure windows could often be defined more clearly based on documented travel histories and identifiable transmission sources outside Guangdong. Therefore, we analyzed inter-provincial cases separately, and yielded a median incubation period of 5.7 days (95% CI 4.7–6.7), with the 95% range spanning from 2.6 (95% CI 1.6–4.5) to 12.4 (95% CI 8.6–16.0) days. Alternatively, inter-provincial cases may represent a subset of persons with longer incubation periods. We further analyzed intra-provincial cases (n = 296), and the median incubation period is estimated to be 5.3 days (95% CI 4.9–5.7), with a 95% range of 2.5 (95% CI 2.2–2.9) to 11.2 (95% CI 9.9–12.5) days. Sensitivity analyses using other commonly used parameterizations of the incubation period (gamma, Weibull, and Erlang distributions) produced similar estimates. Full results of these sensitivity analyses are presented in Table 3.

**Table 3:** Percentiles of the incubation period for CF from selected sensitivity analyses.

Analysis	Incubation Period (95% CI), d				
	2.5th Percentile	25th Percentile	50th Percentile	75th Percentile	97.5th Percentile
Log-normal (n = 400)	2.5 (2.2–2.9)	4.1 (3.8–4.5)	5.4 (5.0–5.7)	6.9 (6.5–7.4)	11.4 (10.1–12.5)
Short-term exposure (n = 42)	2.4 (1.9–3.2)	4.1 (3.5–4.8)	5.4 (4.8–6.1)	7.1 (6.3–7.9)	11.9 (9.5–14.2)
Intra-provincial (n = 296)	2.5 (2.2–2.9)	4.1 (3.8–4.5)	5.3 (4.9–5.7)	6.9 (6.3–7.4)	11.2 (9.9–12.5)
Inter-provincial (n = 104)	2.6 (1.6–4.5)	4.3 (3.3–5.8)	5.7 (4.7–6.7)	7.4 (6.3–8.4)	12.4 (8.6–16.0)
Gamma (n = 400)	2.3 (1.9–2.7)	4.2 (3.8–4.5)	5.5 (5.1–5.9)	7.1 (6.6–7.6)	10.9 (9.7–11.9)
Weibull (n = 400)	1.7 (1.4–2.0)	4.1 (3.8–4.5)	5.7 (5.3–6.1)	7.4 (6.8–7.9)	10.6 (9.5–11.5)
Erlang (n = 400)	2.3 (1.9–2.6)	4.2 (3.8–4.5)	5.5 (5.1–5.9)	7.1 (6.6–7.6)	10.9 (10.1–12.0)

CF = Chikungunya fever.

Using the log-normal model, we estimated the incubation period of CF in four subsets. The primary analysis included all cross-city cases (n = 400), followed by analyses restricted to cases with short-term exposure windows (n = 42), intra-provincial cases (n = 296), and inter-provincial cases (n = 104). Three alternative distributions (gamma, Weibull, and Erlang) were fitted to the full dataset to assess the robustness of the estimates. The median incubation period and key quantiles (2.5th, 25th, 75th, and 97.5th percentiles) were estimated for each model, along with their bootstrapped 95% CIs.

To evaluate the adequacy of the 12-day monitoring policy, we further estimated the probability of symptom onset within 12 days using the fitted incubation-period distributions. Based on the fitted incubation-period distributions, the vast majority of cases were expected to develop symptoms within 12 days after exposure. Under the log-normal model, 98.2% (95%CI 96.7%–99.3%) of cases were estimated to become symptomatic within 12 days, corresponding to a residual risk of 1.8% (95%CI 0.7%–3.3%): after a 12-day observation period. Similar estimates were obtained using alternative distributions, with the probability of symptom onset within 12 days ranging from 98.9% to 99.5%. These results indicate that only a small proportion of cases would be expected to develop symptoms beyond 12 days (Table 4).

**Table 4:** Estimated proportion of symptom onset within 12 days after exposure.

Analysis	Incubation Period (95% CI), d	
	Probability ≤12 days (95% CI)	Residual risk >12 days
Log-normal (n = 400)	98.2% (96.7–99.3)	1.8% (0.7–3.3)
Short-term exposure (n = 42)	97.8% (94.6–99.7)	2.2% (0.3–5.4)
Intra-provincial (n = 296)	98.4% (96.9–99.5)	1.6% (0.5–3.1)
Inter-provincial (n = 104)	97.4% (92.4–100.0)	2.6% (0.0–7.6)
Gamma (n = 400)	98.9% (97.6–99.7)	1.1% (0.3–2.4)
Weibull (n = 400)	99.5% (98.4–99.9)	0.5% (0.1–1.6)
Erlang (n = 400)	98.9% (97.5–99.6)	1.1% (0.4–2.5)

CF = Chikungunya fever.

The estimates were derived from the fitted accelerated failure time model using interval-censored exposure data. The probability of the onset of symptoms within 12 days after exposure and the residual risk were calculated from the estimated distribution of incubation periods. Corresponding 95% CIs were obtained via bootstrap resampling.

## Discussion

Based on the data from the 2025 outbreak of CF in Guangdong Province, in this study, we described the epidemiological characteristics and mobility patterns of the cross-city cases for the first time. Using the well-defined exposure periods of these mobile cases, we estimated that the median incubation period of this CF was 5.4 days (95% CI 5.0–5.7 days), providing more robust estimates regarding the ECSA genotype of CHIKV under real-world epidemic conditions.

Guangdong Province, located in southern China, has a warm and humid climate year-round, which favors the widespread distribution of *A. aegypti* and *A. albopictus* mosquitoes and lays the basis for the outbreak of this epidemic [26–28]. As Guangdong Province is an important economic hub, frequent business interactions and migrant labor flows further facilitate the rapid spread of the disease [29]. Because of better transportation connectivity and global climate change, CHIKV may trigger new outbreaks in more areas of Guangdong and even in other provinces with a similar climate in the future [30,31], such as Guangxi, Fujian, and Yunnan. Therefore, researchers should focus on the cross-city cases and determine their transmission patterns for establishing targeted measures to prevent future outbreaks.

To characterize the cross-city cases, we first compared these cases with local cases in Foshan city of Guangdong Province [20]. The epidemiological characteristics revealed many differences between them, especially regarding age, gender, and occupation. Some studies reported that individuals who were 65 years old or older had the highest incidence of infection, which was consistent with the findings of Foshan city. In contrast, the age group of 15–24, 25–34 and 35–44 years accounted for a higher percentage of cross-city cases in our study, whereas the age group of more than 65 years accounted for a smaller percentage of cases (S1 Table). This discrepancy probably reflects low mobility among the elderly rather than differences in immunity among age groups [32,33]. Occupational distribution also differed considerably: cross-city cases included a higher proportion of students, general staff/workers and farmers, whereas they included a lower proportion of business/service workers, homemakers/unemployed individuals and retirees. This pattern may be attributed to contextual and behavioral factors. The outbreak coincided with summer vacation, a period during which many students return home or travel, and young farmers visit their families in Guangdong. General staff and workers frequently engage in cross-city work-related activities or business trips. In contrast, homemakers, unemployed individuals, and retirees may be less mobile due to economic constraints [33], while business/service workers usually manage their business without traveling extensively. Additionally, cross-city cases included a higher proportion of male which reflected their greater engagement in outdoor and mobile activities [34]. The differences between cross-city cases and local cases in Foshan city highlighted the significance of adjusting prevention strategies tailored to the specific risk of exposure different demographic and occupational groups. Special attention should be paid to the prevention of cross-city epidemics among individuals aged 15–44 years old and among key occupational groups such as students, general office workers, and farmers.

For intra-provincial cases, Foshan was the predominant source of infection (245 cases; 82.8%), followed by Guangzhou (35 cases; 11.8%), which aligned with the overall rank of case numbers. Foshan is a major city in the Pearl River Delta Economic Zone with a population of over 9.62 million and experiences significant population efflux. Data from a migration data platform [35] revealed that the primary destinations for travelers from the districts Shunde, Chancheng, and Nanhai in Foshan were the provinces of Guangxi, Hunan, and Jiangxi, and the cities of Guangzhou, Zhaoqing, and Zhongshan in Guangdong. The consistency between these population outflow patterns and the case spillover data aligns with previous findings demonstrating that a higher total importation risk value predicted a higher level of COVID-19 transmission [36] and highlights the close relationship between the spread of the disease and population mobility. These observations suggest that better clinical awareness and public health messaging are needed in these high-risk receiving areas, particularly in Guangxi, Hunan, and Jiangxi.

Patient mobility across administrative boundaries to access healthcare is a common response to disparities in the distribution of healthcare resources [37]. By analyzing the trajectories of cross-city cases, we found that many patients traveled to Guangzhou—the provincial medical hub—shortly after the onset of symptoms. A significant number of patients also sought healthcare across provincial lines. In China, healthcare system planning and sustainability are managed at the provincial level. Inter-provincial cases have lower reimbursement rates and incur additional costs for transportation and accommodation are incurred if patients return to their domicile [38,39]; this problem highlights the need to enhance the efficiency of the system. These mobility patterns reveal the imbalance between healthcare supply and demand, providing macro-level evidence for optimizing resource allocation. Although policies like direct medical insurance settlement and the “integrated healthcare system” project have reduced financial burdens to some extent and improved coordination [40,41], out-of-pocket expenses remain high for inter-provincial patients. Therefore, targeted government subsidies are therefore necessary to mitigate these disparities and advance healthcare equity.

The incubation period needs to be accurately estimated for inferring the timing and location of infection, modeling disease progression, and evaluating the efficacy of control measures [42–44]. However, the incubation periods of CF are nonspecific, based on rough estimated time intervals or small sample data [16,45,46]. In this study, we estimated the incubation period using detailed documents of 400 cross-city cases for the novel outbreak of CF that occurred in Guangdong Province in 2025. The median incubation period of CF in our study was estimated to be 5.4 days, which is broadly consistent with findings from previous chikungunya outbreaks reported in other regions. Earlier studies have generally reported median incubation periods ranging from approximately 3–7 days [47,48], depending on the study design and exposure assessment. For example, analyses of outbreaks in the Indian Ocean region have suggested median incubation periods of around 4–7 days [49], while a systematic review based on pooling individual case data from multiple published studies reported slightly shorter estimates of average 3 days [16]. Our estimate falls within this range, suggesting that the incubation characteristics observed in Guangdong are comparable to those reported in other endemic or epidemic settings. Moreover, these findings provide quantitative support for the use of a 12-day observation or monitoring period in the management of chikungunya exposure [50]. According to our model-based estimates, approximately 98.2%–99.5% of symptomatic infections would occur within 12 days after exposure, leaving a residual risk of about 0.5%–1.8% thereafter. Although a small proportion of cases may develop symptoms beyond this period, a 12-day monitoring strategy would capture the vast majority of infections while maintaining a reasonable balance between public health protection and operational feasibility. However, longer monitoring periods might be justified in extreme cases. We expect 179 in 10,000 infected individuals to develop symptoms after the end of a 12-day monitoring period (Table 4). Moreover, the potential for asymptomatic infection suggests that the actual risk may be higher. It is essential to weigh the costs of extending active monitoring or mosquito vector control against the potential costs of failing to identify a symptomatic case. However, high-risk scenarios, such as cross-city family travel with a subsequent diagnosis, need prolonged, intensified surveillance and vector control beyond 12 days to prevent further spread. Beyond informing the duration of active monitoring, a well-characterized incubation-period distribution provides an essential temporal framework for outbreak investigation and response. In particular, it helps define the most plausible exposure window during epidemiological investigations, thereby improving the efficiency of contact tracing and source identification in settings with frequent population mobility. Moreover, accurate incubation estimates enable more reliable interpretation of epidemic curves and the timing of intervention effects, which is critical for assessing the effectiveness of control measures such as vector control and mobility restrictions.

Our findings collectively revealed that inter-city surveillance and early warning systems for mosquito-borne diseases need to be intensified, particularly among the high-risk populations evaluated in our study. Integrating these judgments and estimates presented here can support public health authorities in developing rational, evidence-based control strategies for CF, which ultimately facilitates earlier case detection and more effective containment of local outbreak.

### Limitations

This study had several limitations. First, the absence of comprehensive demographic data on cross-city movement prevented the calculation of incidence rates among mobile populations and comparisons with overall incidence, thereby restricting further analysis of transmission dynamics, which is essential for formulating targeted policies and control measures. Second, for cases with uncertain exposure or dates of symptom onset, we applied conservative assumptions, although unaccounted inaccuracies may remain in the data. In addition, our analysis relied exclusively on reported confirmed cases, which may over-represent severe or hospitalized infections, potentially biasing the estimated incubation period relative to milder or asymptomatic infections. Third, the observed pattern of patients returning to their hometowns for medical care need to be further investigated. Future studies incorporating patient surveys could offer valuable insights into individual motivations, enabling a multi-scale analytical approach that integrates both macro-level flow trends and micro-level behavioral factors. Such studies may be more informative for cross-jurisdictional resource allocation and public health planning.

## Supporting information

S1 Table Comparison of demographic characteristics between early cases in Foshan and cross-city cases. (XLSX)

S2 Table Comparison of exposure window lengths between inter-provincial and intra-provincial cases. (XLSX)

S1 Data Raw data used for the analysis of incubation period estimation. (Provided as an Excel file).
(XLSX)

## References

[1] Bettis AA, L’Azou Jackson M, Yoon I-K, Breugelmans JG, Goios A, Gubler DJ, et al. The global epidemiology of chikungunya from 1999 to 2020: A systematic literature review to inform the development and introduction of vaccines. PLoS Negl Trop Dis. 2022;16(1):e0010069. doi: 10.1371/journal.pntd.0010069 35020717 PMC8789145

[2] Silva LA, Dermody TS. Chikungunya virus: Epidemiology, replication, disease mechanisms, and prospective intervention strategies. J Clin Invest. 2017;127(3):737–49. doi: 10.1172/JCI84417 28248203 PMC5330729

[3] Paixão ES, Rodrigues LC, Costa M da CN, Itaparica M, Barreto F, Gérardin P, et al. Chikungunya chronic disease: A systematic review and meta-analysis. Trans R Soc Trop Med Hyg. 2018;112(7):301–16. doi: 10.1093/trstmh/try063 30007303

[4] Kang H, Auzenbergs M, Clapham H, Maure C, Kim J-H, Salje H, et al. Chikungunya seroprevalence, force of infection, and prevalence of chronic disability after infection in endemic and epidemic settings: A systematic review, meta-analysis, and modelling study. Lancet Infect Dis. 2024;24(5):488–503. doi: 10.1016/S1473-3099(23)00810-1 38342105

[5] Bellone R, Lechat P, Mousson L, Gilbart V, Piorkowski G, Bohers C, et al. Climate change and vector-borne diseases: A multi-omics approach of temperature-induced changes in the mosquito. J Travel Med. 2023;30(4):taad062. doi: 10.1093/jtm/taad062 37171132

[6] Ni J, Li Z, Hu X, Zhou H, Gong Z. Chikungunya’s global rebound and Asia’s growing vulnerability: Implications for integrated vector control and pandemic preparedness. Biosci Trends. 2025;19(4):404–9. doi: 10.5582/bst.2025.01239 40790814

[7] Levi LI, Vignuzzi M. Arthritogenic alphaviruses: A worldwide emerging threat?. Microorganisms. 2019;7(5):133. doi: 10.3390/microorganisms7050133 31091828 PMC6560413

[8] Renault P, Solet JL, Sissoko D, Balleydier E, Larrieu S, Filleul L. A major epidemic of chikungunya virus infection on Reunion Island, France, 2005-2006. The American Journal of Tropical Medicine and Hygiene. 2007;77(4):727–31. 17978079

[9] World Health Organization. Pathogens prioritization: a scientific framework for epidemic and pandemic research preparedness 2024. https://www.who.int/publications/m/item/pathogens-prioritization-a-scientific-framework-for-epidemic-and-pandemic-research-preparedness

[10] Liao CX, Du HP, Wang B, Lyu J, Li LM. Epidemiology, clinical characteristics and prevention strategies of Chikungunya fever. Zhonghua liu xing bing xue za zhi = Zhonghua liuxingbingxue zazhi. 2025;46(8):1468–72. doi: 10.3760/cma.j.cn112338-20250726-00527 40854778

[11] Wei Y, Wang J, Song W, Xiu C, Ma L, Pei T. Spread of COVID-19 in China: Analysis from a city-based epidemic and mobility model. Cities. 2021;110:103010. doi: 10.1016/j.cities.2020.103010 33162634 PMC7598765

[12] Nishiura H, Lee H-W, Cho S-H, Lee W-G, In T-S, Moon S-U, et al. Estimates of short- and long-term incubation periods of Plasmodium vivax malaria in the Republic of Korea. Trans R Soc Trop Med Hyg. 2007;101(4):338–43. doi: 10.1016/j.trstmh.2006.11.002 17204297

[13] Li Y, Jiang S, Zhang M, Li Y, He J, Yang Z, et al. An Outbreak of Chikungunya Fever in China - Foshan City, Guangdong Province, China, July 2025. China CDC weekly. 2025;7(32):1064–5. doi: 10.46234/ccdcw2025.172 40837139 PMC12361914

[14] Mohan A, Kiran DHN, Manohar IC, Kumar DP. Epidemiology, clinical manifestations, and diagnosis of Chikungunya fever: Lessons learned from the re-emerging epidemic. Indian J Dermatol. 2010;55(1):54–63. doi: 10.4103/0019-5154.60355 20418981 PMC2856377

[15] Pialoux G, Gaüzère BA, Jauréguiberry S, Strobel M. Chikungunya, an epidemic arbovirosis. The Lancet Infectious Diseases. 2007;7(5):319–27. doi: 10.1016/s1473-3099(07)70107-x 17448935

[16] Rudolph KE, Lessler J, Moloney RM, Kmush B, Cummings DAT. Incubation periods of mosquito-borne viral infections: A systematic review. Am J Trop Med Hyg. 2014;90(5):882–91. doi: 10.4269/ajtmh.13-0403 24639305 PMC4015582

[17] Chen L, Xing Y, Zhang Y, Xie J, Su B, Jiang J, et al. Long-term variations of urban-Rural disparities in infectious disease burden of over 8.44 million children, adolescents, and youth in China from 2013 to 2021: An observational study. PLoS Med. 2024;21(4):e1004374. doi: 10.1371/journal.pmed.1004374 38607981 PMC11014433

[18] Yu L-J, Ji P-S, Ren X, Wang Y-H, Lv C-L, Geng M-J, et al. Inter-city movement pattern of notifiable infectious diseases in China: A social network analysis. Lancet Reg Health West Pac. 2024;54:101261. doi: 10.1016/j.lanwpc.2024.101261 39759426 PMC11700286

[19] National Health Commission of the People’s Republic of China. Diagnostic Criteria for Chikungunya Fever. https://www.ndcpa.gov.cn/jbkzzx/crb/common/content/content_1656311979820519424.html

[20] Zhang M, Li Y, Huang X, Liu M, Jiang S, Zeng B, et al. Epidemiological characteristics and transmission dynamics of the early stage Chikungunya fever outbreak in Foshan City, Guangdong Province, China in 2025. Infectious Diseases of Poverty. 2025;14(1):93. doi: 10.1186/s40249-025-01364-y 40936090 PMC12424219

[21] Reich NG, Lessler J, Cummings DAT, Brookmeyer R. Estimating incubation period distributions with coarse data. Stat Med. 2009;28(22):2769–84. doi: 10.1002/sim.3659 19598148

[22] Tuite AR, Watts AG, Khan K, Bogoch II. Countries at risk of importation of chikungunya virus cases from Southern Thailand: A modeling study. Infect Dis Model. 2019;4:251–6. doi: 10.1016/j.idm.2019.09.001 31667444 PMC6812318

[23] España G, Grefenstette J, Perkins A, Torres C, Campo Carey A, Diaz H, et al. Exploring scenarios of chikungunya mitigation with a data-driven agent-based model of the 2014-2016 outbreak in Colombia. Sci Rep. 2018;8(1):12201. doi: 10.1038/s41598-018-30647-8 30111778 PMC6093909

[24] Chan M, Johansson MA. The incubation periods of Dengue viruses. PLoS One. 2012;7(11):e50972. doi: 10.1371/journal.pone.0050972 23226436 PMC3511440

[25] Raza A, Arif MS, Rafiq M. A reliable numerical analysis for stochastic dengue epidemic model with incubation period of virus. Adv Differ Equ. 2019;2019(1). doi: 10.1186/s13662-019-1958-y

[26] Xie W, Zhang H, Ni Y, Peng Y. Contrasting diversity and composition of human colostrum microbiota in a maternal cohort with different ethnic origins but shared physical geography (Island Scale). Front Microbiol. 2022;13:934232. doi: 10.3389/fmicb.2022.934232 35903466 PMC9315263

[27] Lai S, Huang Z, Zhou H, Anders KL, Perkins TA, Yin W, et al. The changing epidemiology of dengue in China, 1990-2014: A descriptive analysis of 25 years of nationwide surveillance data. BMC Med. 2015;13:100. doi: 10.1186/s12916-015-0336-1 25925417 PMC4431043

[28] Yin Y, Xu Y, Su L, Zhu X, Chen M, Zhu W, et al. Epidemiologic investigation of a family cluster of imported ZIKV cases in Guangdong, China: Probable human-to-human transmission. Emerg Microbes Infect. 2016;5(9):e100. doi: 10.1038/emi.2016.100 27599469 PMC5113051

[29] Welch SB, Kulasekere DA, Prasad PVV, Moss CB, Murphy RL, Achenbach CJ. The interplay between policy and COVID-19 outbreaks in South Asia: Longitudinal trend analysis of surveillance data. JMIR Public Health and Surveillance. 2021;7(6):e24251. doi: 10.2196/24251 PMC8213065 34081593

[30] Gao Y, Goonawardane N, Ward J, Tuplin A, Harris M. Multiple roles of the non-structural protein 3 (nsP3) alphavirus unique domain (AUD) during Chikungunya virus genome replication and transcription. PLoS Pathog. 2019;15(1):e1007239. doi: 10.1371/journal.ppat.1007239 30668592 PMC6358111

[31] Qiu S, Guo J, Li P, Li P, Du X, Hao R, et al. Source-tracking of the Chinese Chikungunya viruses suggests that Indian subcontinent and Southeast Asia act as major hubs for the recent global spread of Chikungunya virus. Virol J. 2021;18(1):203. doi: 10.1186/s12985-021-01665-2 34635129 PMC8507386

[32] de Morais Alves Barbosa Oliveira R, Kalline de Almeida Barreto F, Praça Pinto G, Timbó Queiroz I, Montenegro de Carvalho Araújo F, Wanderley Lopes K. Chikungunya death risk factors in Brazil, in 2017: A case-control study. PLoS One. 2022;17(4):e0260939. doi: 10.1371/journal.pone.0260939 PMC8989201 35389992

[33] Zhao P, Yu Z. Investigating mobility in rural areas of China: Features, equity, and factors. Transport Policy. 2020;94:66–77. doi: 10.1016/j.tranpol.2020.05.008

[34] Abdulmoghni RT, Al-Ward AH, Al-Moayed KA, Al-Amad MA, Khader YS. Incidence, trend, and mortality of human exposure to rabies in Yemen, 2011-2017: observational study. JMIR Public Health and Surveillance. 2021;7(6):e27623. doi: 10.2196/27623 PMC8277343 34156339

[35] Crowd migration situation awareness platform 2025. https://lbs.qq.com/bigdata/migration/

[36] Liu J, Hao J, Sun Y, Shi Z. Network analysis of population flow among major cities and its influence on COVID-19 transmission in China. Cities. 2021;112:103138. doi: 10.1016/j.cities.2021.103138 33564205 PMC7862886

[37] Yang J, Yan B, Fan S, Ni Z, Yan X, Xiao G. Cross-provincial inpatient mobility patterns and their determinants in China. BMC Health Serv Res. 2024;24(1):1004. doi: 10.1186/s12913-024-11436-8 39210361 PMC11363524

[38] Gu H, Wu D. Connotation and strategic conceptualization of the high-quality development of the essential healthcare security system during the 14th five-year plan period. J Manage World. 2021;37(09):158–67.

[39] Wang X, Li X, Fan J. Current situation and trend analysis of inpatients in grade III hospitals seeking medical treatment across provinces and places from 2015 to 2020. 2023;21(04):51–3.

[40] Tao W, Zeng Z, Dang H, Lu B, Chuong L, Yue D, et al. Towards universal health coverage: Lessons from 10 years of healthcare reform in China. BMJ Global Health. 2020;5(3):e002086. doi: 10.1136/bmjgh-2019-002086 PMC7103824 32257400

[41] Zhang C, Guo M, Liu Y, Liu Y. A preliminary study on the construction and effect of the coordination mechanism of cross-provincial treatment direct reimbursement services. China Health Insurance. 2021;12:27–32.

[42] Fraser C, Riley S, Anderson RM, Ferguson NM. Factors that make an infectious disease outbreak controllable. Proc Natl Acad Sci U S A. 2004;101(16):6146–51. doi: 10.1073/pnas.0307506101 15071187 PMC395937

[43] Lessler J, Brookmeyer R, Perl TM. An evaluation of classification rules based on date of symptom onset to identify health-care associated infections. Am J Epidemiol. 2007;166(10):1220–9. doi: 10.1093/aje/kwm188 17702972

[44] Lessler J, Brookmeyer R, Reich NG, Nelson KE, Cummings DAT, Perl TM. Identifying the probable timing and setting of respiratory virus infections. Infect Control Hosp Epidemiol. 2010;31(8):809–15. doi: 10.1086/655023 20569117

[45] Lessler J, Reich NG, Brookmeyer R, Perl TM, Nelson KE, Cummings DAT. Incubation periods of acute respiratory viral infections: A systematic review. Lancet Infect Dis. 2009;9(5):291–300. doi: 10.1016/S1473-3099(09)70069-6 19393959 PMC4327893

[46] Reich NG, Perl TM, Cummings DAT, Lessler J. Visualizing clinical evidence: Citation networks for the incubation periods of respiratory viral infections. PLoS One. 2011;6(4):e19496. doi: 10.1371/journal.pone.0019496 21559339 PMC3084881

[47] De Valk H, Leparc-Goffart I, Paty MC, Reusken C, van den Kerkhof H, Braks M. Autochthonous cases of chikungunya fever on the Caribbean island, Saint Martin. ECDC. 2013.

[48] Staples JE, Powers AM. CDC Yellow Book: Health Information for International Travel 2026.

[49] Lahariya C, Pradhan SK. Emergence of chikungunya virus in Indian subcontinent after 32 years: A review. J Vector Borne Dis. 2006;43(4):151–60. 17175699

[50] Chinese Center for Disease Control and Prevention. Technical Guidelines for Prevention and Control of Chikungunya Fever 2025 [cited 2025-09-25]. https://www.chinacdc.cn/jkyj/crb2/qt/jkkyr/jswj_jkkyr/202507/U020250730498723615270.pdf

